# Aging and the perception of tactile speed

**DOI:** 10.1038/s41598-022-09493-2

**Published:** 2022-03-30

**Authors:** J. Farley Norman, Jerica R. Eaton, McKenzie L. Gunter, Maheen Baig

**Affiliations:** 1 Department of Psychological Sciences, Ogden College of Science and Engineering, Western Kentucky University, 1906 College Heights Blvd. #22030, Bowling Green, KY 42101-2030 USA; 2 Center for Applied Science in Health and Aging, Western Kentucky University, Bowling Green, KY 42101-2030 USA

**Keywords:** Perception, Human behaviour

## Abstract

Eighteen younger and older adults (mean ages were 20.4 and 72.8 years, respectively) participated in a tactile speed matching task. On any given trial, the participants felt the surfaces of rotating standard and test wheels with their index fingertip and were required to adjust the test wheel until its speed appeared to match that of the standard wheel. Three different standard speeds were utilized (30, 50, and 70 cm/s). The results indicated that while the *accuracy* of the participants’ judgments was similar for younger and older adults, the *precision* (i.e., reliability across repeated trials) of the older participants’ judgments deteriorated significantly relative to that exhibited by the younger adults. While adverse effects of age were obtained with regards to both the precision of tactile speed judgments and the participants’ tactile acuity, there was nevertheless no significant correlation between the older adults’ tactile acuities and the precision of their tactile speed judgments.

## Introduction

In our everyday human behavior, we pick up solid environmental objects on a frequent basis. Of course, much of what we know and learn about such objects (e.g., their shape and surface material) depends upon vision^1,2^. However, our sense of touch is also quite capable. When we actively explore and manipulate solid objects, our fingers necessarily slide across their surfaces, producing relative movement between fingertips and object. About 60 years ago, Gibson^3^ showed that this relative movement permits shape recognition at levels of accuracy far higher than is obtained with no relative motion (i.e., with passive touch). In addition, the pioneering research of Katz^4^ demonstrated that a sufficient speed of relative movement between fingertip and surface is needed for accurate material perception and recognition (e.g., determining whether a surface is composed of glass, metal, wood, paper, stone, fabric, etc.). Even our ability to grip and hold objects successfully depends upon a sensitivity to tactile motion. For example, if unexpected tactile motion relative to an object surface (i.e., slip) is detected during the initial grasp of an object, the grip is automatically strengthened^5^. Given the importance of tactile motion for everyday life activities, it is perhaps not surprising that we can effectively judge both the direction^6–9^ and speed^10–15^ of a tactile motion stimulus.

It has been known since 2003 that aging results in a significant deterioration in the ability to visually judge speed^16–19^. As an example, consider the original study by Norman, Ross, Hawkes, and Long^16^. Younger and older adults performed a 2-AFC (2-alternative spatial forced choice) task and were required to indicate on any particular trial which of two strips of moving points moved faster^20^. Negative effects of age (i.e., increased speed discrimination thresholds for the older adults) occurred for each of the three standard speeds (1.22, 5.48, and 24.34 deg/s); at the medium standard speed (where overall performance was best) the speed discrimination thresholds of the older adults (mean age was 72.6 years) were 71% higher than those obtained for the younger adults (mean age was 21.8 years).

It has been known for about 40 years^21,22^ that neurons in cortical area MT are selective and tuned for the speed of visual motion. It is important and interesting to note at this point that these speed-sensitive MT neurons respond to both visual and tactile motion^23–26^. Since (1) aging adversely affects visual speed perception and discrimination^16–19^ and (2) the cortical mechanisms responsible for perceiving visual speed also respond to tactile speed^23–26^, it seems likely that aging also negatively affects tactile speed discrimination. The purpose of our current experiment is straightforward, to test this possibility—no previous study has ever evaluated the potential effects of increased age upon tactile speed perception.

## Methods

### Apparatus and experimental stimuli

Like other previous investigators^12–15^, we used an apparatus consisting of rotating wheels. The wooden standard and test wheels (1.8 cm thick plywood) were 20.2 cm in diameter (63.5 cm circumference), and were separated spatially by 9.6 cm. The participants used the fingertip of their index finger to feel the natural wooden textured surface (outer circumference) of the wheels through rectangular apertures^11,14,15^. The wheels were driven by variable-speed DC motors. Since the participants’ fingertips were quite small relative to the circumference of the wheels (approximately 1.5 cm versus 63.5 cm), the participants could not feel the curvature—the wooden surface effectively translated underneath the participants’ fingertip. An Apple MacBook computer was used to randomly order the standard wheel speeds and record the participants’ responses. The participants’ tactile acuity was measured using standard JVP Domes^27–31^ (i.e., tactile gratings).

### Procedure

On any given trial, the participants initially felt the surface of the standard wheel and were then required to adjust the speed of the test wheel until its speed matched (i.e., felt identical to) that of the standard. The participants gently touched the moving surfaces^13^ and used the same fingertip to alternately feel the standard and test wheels. The participants were allowed as much time as they needed to make their matching adjustments (the participants were allowed to alternate between the two wheels until they were satisfied that the two speeds felt equivalent). There were a total of three standard speeds (30, 50, and 70 cm/s). Five trials were conducted for each of the three standard speeds—the order of the resulting 15 trials were run in a completely random order (which was different for every participant). At the beginning of each trial, the experimenter set the initial speed of the test wheel to a random computer-determined value. During the experiment, the participants wore both opaque goggles (to prevent the participants from seeing the rotating wheels) and ear muffs (34 db noise reduction) to eliminate the sound of the motors.

In measuring the participants’ tactile acuity, we followed procedures used in previous research^30,31^. Participants first completed a block of 40 trials with a large groove width (e.g., 3 mm for the younger adults); the task was to determine on any given trial whether the grooves of the tactile grating^27,29^ were applied (to the distal pad of their index finger) in a direction that was parallel or perpendicular to the long axis of their finger. Subsequent blocks were conducted with smaller and smaller groove widths until the participants’ discrimination accuracy dropped below a dʹ value^32^ of 1.35. Linear interpolation^27^ was then used to determine the participants’ final grating orientation threshold (i.e., the groove width needed to discriminate grating orientation with a dʹ of 1.35). Because it is well known that aging is associated with an overall reduction in tactile acuity^30,31,33,34^, the older participants initially judged tactile gratings (in the first block) with a larger groove width of 4 or 5 mm.

### Participants

In our most recent analogous study of aging and visual speed matching^19^, a total of 14 participants (7 younger and 7 older) gave sufficient power to detect a significant effect of increased age upon precision. In the current investigation, we therefore recruited 14 naive participants (7 younger adults and 7 older adults). The four coauthors (JFN, JRE, MLG, and MB) also participated in the experiment^12,13,24^, bringing the total number of younger and older participants to 10 and 8, respectively. The mean ages of the younger and older participants were 20.4 years (ages ranged from 20 to 22 years, sd = 0.7) and 72.8 years (ages ranged from 60 to 84 years, sd = 7.7), respectively. Older adults in their 60’s, 70’s, and 80’s were included so that we could evaluate whether variations in chronological age within the older group affect performance; for example, it would be important to determine whether the tactile speed judgments of older old adults are as precise as those made by younger old adults. The study was approved by the Institutional Review Board of Western Kentucky University, and each participant signed an informed consent document prior to testing. Our research was carried out in accordance with the Code of Ethics of the World Medical Association (Declaration of Helsinki).

## Results

**Figure 1:**
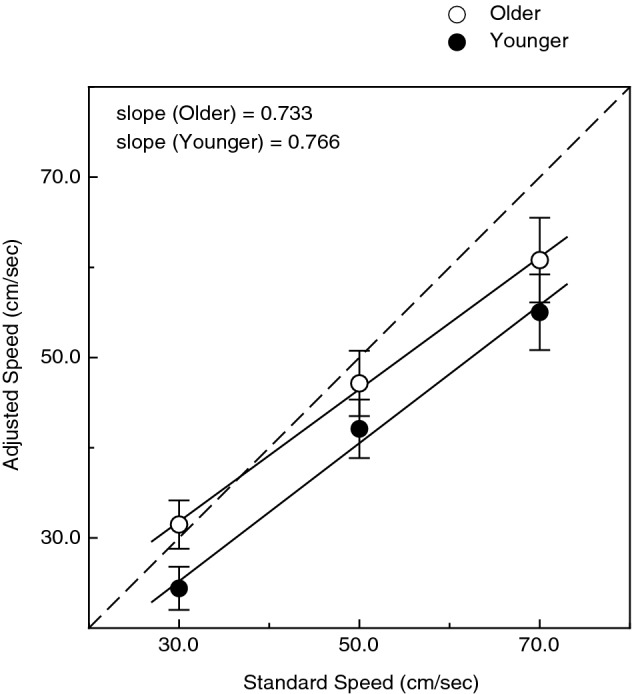
The younger and older participants’ adjusted test speeds as a function of the standard speed. Accurate performance would be indicated by the dashed line. The younger and older participants’ results are indicated by filled and open circles, respectively. The error bars indicate ± 1 SE. The best fitting linear regressions are shown, as well as the slopes of those regressions. One can see that as the standard speed is increased, the perceived speed increases at essentially the same rate for younger and older adults.

**Figure 2:**
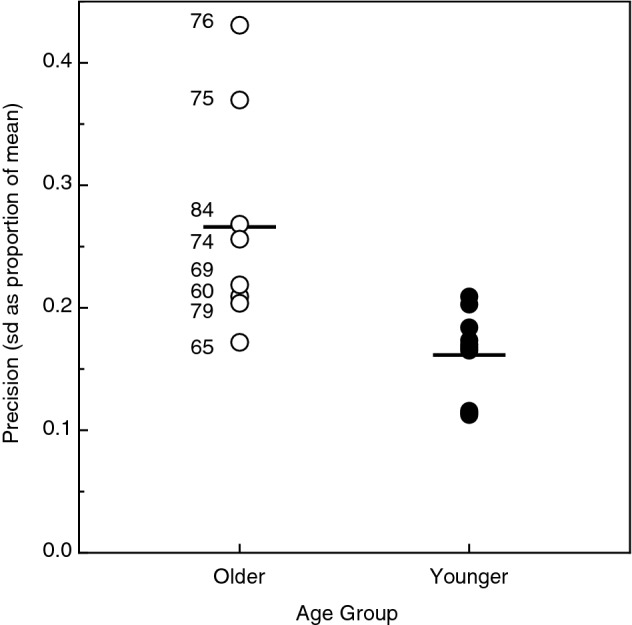
Estimates of precision (standard deviation of repeated judgments as a proportion of the mean) for each of the 18 individual younger and older participants. The individual older participants’ precision values are indicated by open circles, while those for the younger participants are indicated by filled circles. The horizontal lines indicate the mean for each age group. The individual (chronological) ages of the older adults are indicated beside their respective data points.

The results concerning accuracy and precision are shown in Figs. 1 and 2, respectively. It is readily apparent from an inspection of Fig. 1 that as the speed of the standard wheel increased, the perceived speed also increased (F(2, 32) = 100.0, p < 0.000001; η^2^_p_ = 0.86) in a linear manner similar to that observed by Delhaye et al.^35^. In contrast, there was no significant main effect of age (F(1, 16) = 1.8, p = 0.20; η^2^_p_ = 0.10) nor was there an age × standard speed interaction (F(2, 32) = 0.12, p = 0.89; η^2^_p_ = 0.007); therefore, the effect of the variations in standard speed was similar for both younger and older adults.

The precision of the individual participants’ speed-matching judgments is shown in Fig. 2. The results have been collapsed across standard speed since a 2-way split-plot analysis of variance demonstrated that (1) there was no effect of standard speed upon the precision of the participants’ repeated judgements (F(2, 32) = 1.3, p = 0.28; η^2^_p_ = 0.08) and 2) no age × standard speed interaction (F(2, 32) = 1.5, p = 0.23; η^2^_p_ = 0.09). The horizontal lines in Fig. 2 indicate the mean precision (standard deviation of repeated matching adjustments as a proportion of the mean of those same judgments) for each age group. One can see that while there is variability within each age group, there is nevertheless a significant adverse effect of age upon precision (F(1, 16) = 11.5, p = 0.004; η^2^_p_ = 0.42). While the estimates of precision varied considerably within the older group of adults (left portion of Fig. 2), there was no significant correlation (Pearson r = 0.42, p = 0.31, 2-tailed) between the individual ages of the older adults and their precision values. Even if this correlation had been significant, variations in the chronological ages of the older participants would have accounted for only 17.4% (r^2^ = 0.174) of the variations in their precision estimates.

**Figure 3:**
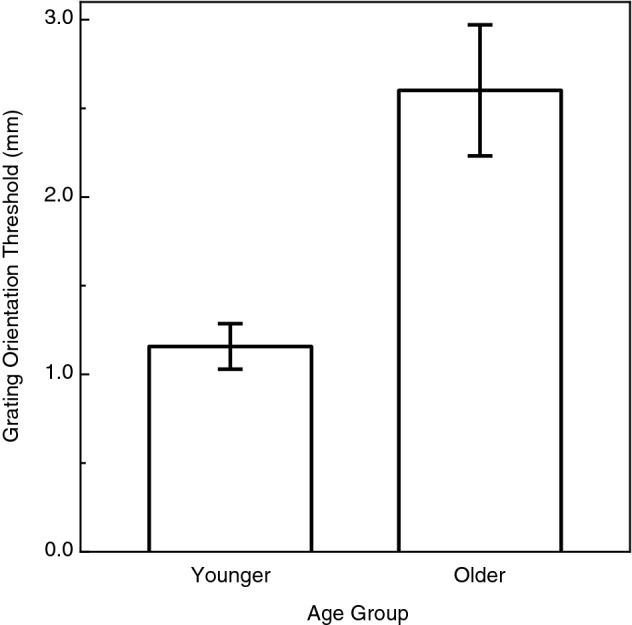
Plots of the younger and older participants’ tactile acuities (i.e., grating orientation thresholds). The error bars indicate ± 1 SE.

The younger and older participants’ tactile acuities (i.e., grating orientation thresholds) are shown in Fig. 3. It is readily apparent from these results that there was a very large adverse effect of increasing age (t(16) = 4.0, p < 0.001; Cohen’s D = 1.9). It is interesting to note in this context, however, that while adverse effects of age were obtained with regards to both the precision of tactile speed judgments (Fig. 2) and the participants’ tactile acuity (Fig. 3), there was nevertheless no significant correlation between the older adults’ tactile acuities and the precision of their tactile speed judgments (Pearson r =  − 0.26, p = 0.53, 2-tailed). Even if this correlation had been significant, variations in our older participants’ tactile acuities would have accounted for only 6.8% (r^2^ = 0.068) of the variation in the precision of their tactile speed-matching judgments. If variations in tactile acuity are not responsible for variations in the ability to judge tactile speed, what could the responsible factor be? One possibility has been suggested by Bennett et al.^36^ from their studies on aging and visual motion perception. These authors concluded that their results were consistent with the idea that older adults possess increased internal noise within the visual system (i.e., increased spontaneous activity of neurons within the visual system, resulting in reduced selectivity of relevant neurons to direction and/or speed of visual motion, etc.). Given the similarities between visual and tactile motion perception (e.g., cortical MT neurons respond to both visual and tactile motion), variations in internal noise within tactile cortical mechanisms could likewise be responsible for the variations in precision exhibited by our participants in Fig. 2.

## Discussion

In our previous study of aging and visual speed matching^19^, the overall standard deviations of repeated judgments for the older adults were 53.2% higher than those obtained for the younger adults (standard deviations were 11.95 and 7.80% of the mean for the older and younger participants, respectively). The size of the analogous effect of age obtained in the current tactile study was similar, but slightly larger—64.8% higher in the older adults (standard deviations were 26.60 and 16.14% of the mean for the older and younger participants, respectively).

As we have seen, aging has large effects upon the precision of both visual^16–19^ and tactile (current study, Fig. 2) judgments of speed. Given the results of recent research, perhaps this behavioral outcome is not altogether surprising. Multiple studies^6,10,12,24^ have now demonstrated that visual and tactile motion are processed by overlapping and interacting mechanisms in cerebral cortex. For example, in the study by Bensmaïa, Killebrew, and Craig^10^ the presence of a task-irrelevant visual drifting sinusoidal grating modified the perceived speed of a tactile motion stimulus (e.g., see Table 1 of Bensmaïa et al.^10^). In this context, it is important to remember that cortical area MT not only responds to visual motion, but tactile motion as well^23–26,37^. If increases in age lead to deteriorations in the ability to precisely judge the speed of visual motion^16–19^ and if cortical mechanisms devoted to motion (such as area MT) are sensitive to both visual and tactile speed, then our current finding of an age-related deterioration in the precision of tactile speed judgments is quite understandable.

If increases in age reduce neuronal selectivity to visual motion^38–42^ and if this functional deficit is caused by a relative lack of GABA activity (reduced inhibition) in senescent visual mechanisms^38–46^, then given the current results (age-related reduction in precision/difference thresholds for tactile motion judgments), one would expect to find an analogous GABA-related reduction in the selectivity of tactile motion-sensitive neurons in old/senescent primates. It remains for future neurophysiological investigations to verify this prediction—i.e., to determine whether an age-related reduction in GABA inhibition does exist for somatosensory neurons sensitive to tactile motion (so that they become less selective). An age-related reduction in cortical inhibition does exist for vision and would be consistent with our current tactile psychophysical results.

## Conclusion

Older adults can make tactile judgments of speed that are as accurate as those of younger adults; nevertheless the tactile judgments of older adults exhibit substantially reduced precision.

## Data Availability

The datasets generated during and/or analyzed during the current study are available from the corresponding author on reasonable request.
